# Single‐port extraperitoneal robot‐assisted simple prostatectomy for benign prostatic hyperplasia: Surgical technique and initial clinical experience

**DOI:** 10.1002/bco2.70273

**Published:** 2026-08-24

**Authors:** Sij Hemal, Samantha H. Rosen, Simon Kim, Joon Yau Leong, Michael Eppler, Patrick Ford, Jacob S. Hershenhouse

**Affiliations:** ^1^ Catherine and Joseph Aresty Department of Urology, USC Institute of Urology, Keck School of Medicine University of Southern California Los Angeles California USA

**Keywords:** benign prostatic hyperplasia, minimally invasive surgery, robotic surgery, simple prostatectomy, single‐port surgery

## 1 INTRODUCTION

Simple prostatectomy via an open, laparoscopic or robotic approach is recommended by both the American Urologic Association (AUA) and European Association of Urology (EAU) guidelines for male lower urinary tract symptoms (LUTS) attributable to benign prostatic hyperplasia (BPH) for large (80–150 cm^3^ [cc]) and very large prostate glands (>150 cc).^1^, ^2^ Laser enucleation using the holmium or thulium laser is also recommended for these gland sizes, but a steep learning curve and the operator‐dependent rates of transient initial incontinence have limited adoption of this treatment modality in the United States. In 2018, the Food and Drug Administration cleared the da Vinci single‐port (SP) system for performing robot‐assisted simple prostatectomy (RASP). Abou Zeinab et al. subsequently described a transvesical (TV) approach with robust clinical outcomes relative to multi‐port RASP, including high rates of same‐day discharge (SDD) and shorter length of stay (LOS) secondary to surgical regionalization.^3^ Furthermore, the excellent long‐term durability and promising outcomes at 1 year following the index operation with sustained improvement in International Prostate Symptom Score (IPSS), quality of life, maximum flow rates, post‐void residual volumes (PVRs) and no reported long‐term stress urinary incontinence (SUI), bladder neck contracture or urethral stenosis has led to the widespread adoption of the SP TV RASP among the SP robotic surgeons in the United States.^4^

The TV approach to RASP has demonstrated several advantages, though certain limitations exist in the anterior/apical adenoma dissection, performance of vesicourethral anastomosis in a narrow working space and the rare risk of an air embolism, arising from pneumovesicum in a valveless port insufflation system. Herein, we describe an alternative SP RASP technique using an extraperitoneal (EP) approach that maintains the benefits of regionalized surgery, may facilitate en bloc anatomic enucleation, simplifies anterior/apical enucleation, accommodates moderate‐to‐large median lobes and achieves a vesicourethral reconstruction that obviates continuous bladder irrigation (CBI).

## 2 PATIENTS AND METHODS

We included all consecutive patients undergoing SP EP RASP performed by a single robotic surgeon (S.H.) at a high‐volume academic medical centre between July 2025 and April 2026. This cohort captures the surgeon's first independently performed SP EP simple prostatectomies and describes the technique and initial outcomes. Baseline demographics, clinical characteristics, perioperative variables and postoperative outcomes were collected in a prospectively maintained database and analysed retrospectively.

Baseline patient characteristics included age at time of surgery, race, ethnicity, body mass index (BMI), Charlson Comorbidity Index (CCI), American Society of Anesthesiologists (ASA) score and relevant comorbidities including hypertension, diabetes mellitus and hyperlipidaemia. Preoperative variables included PSA level, prostate volume and prior biopsy status. Prostate volume was determined from preoperative MRI, ultrasound or CT cross‐sectional imaging that was clinically obtained at the referring urologist's discretion.

Perioperative variables included console time (defined as time from robot docking to undocking), total operative time (defined as time from incision to closure), estimated blood loss (EBL), conversion to multi‐port or open surgery, placement of an additional port (‘plus one’) or drain placement and intraoperative complications classified according to the Clavien–Dindo system.

Short‐term postoperative outcomes included LOS, postoperative haemoglobin levels, transfusion requirements, 30‐ and 90‐day complications according to Clavien–Dindo classification and 30‐day readmission rates. Functional outcomes were assessed at 4‐week and 3‐month follow‐up appointments postoperatively and included return of continence status (defined as being pad‐free or using a single pad for safety) and post‐void residuals at follow‐up.

Given that this cohort represents the surgeon's initial independent experience with the SP EP RASP technique, a learning curve analysis and cumulative sum (CUSUM) methodology was applied to total operative time as the primary metric for performance. The CUSUM value for each sequential case was calculated as the cumulative deviation of that case's operative time from the cohort mean operative time. Operative time trends across the learning curve were further characterized using locally estimated scatterplot smoothing (LOESS).

Descriptive statistics were used to summarize baseline characteristics, perioperative outcomes and postoperative results. Continuous variables are presented as median with interquartile range (IQR) or mean with standard deviation as appropriate. Categorical variables are presented as frequencies and percentages.

## 3 SURGICAL TECHNIQUE

This technique is described with a video legend as a supplementary document. The patient is positioned supine with the table flat, avoiding any Trendelenburg. After sterile preparation and Foley catheter placement, a 3.5‐ to 4‐cm transverse incision is made approximately 6 cm above the pubic symphysis. Dissection proceeds to the anterior rectus sheath, which is incised. The rectus bellies are identified and split in the midline using a tonsil clamp thereby exposing the preperitoneal space developed manually by sliding the surgeon's index finger towards the undersurface of the pubic symphysis, without a space maker balloon. The small incision da Vinci SP access port kit (Intuitive Surgical, Inc.) is then placed and assembled in the created EP space and the robot is docked using the floating dock technique. The EP space is insufflated to 10 mmHg using an 8‐mm AirSeal port (CONMED, Utica, NY, USA) that is inserted into the SP access kit. A ‘plus one’ 5‐mm port was used selectively in early cases with very large glands. The placement of an additional 5‐mm port in the EP space allows bedside suctioning/retraction with a metal Stryker™ suction should the ROSI (VTI, Nashua, NH USA) become obstructed by blood products. The ‘plus one’ 5‐mm port is placed approximately 7‐cm lateral and slightly cranial to the assembled SP access kit into the manually created EP space.

Following docking, SP instrumentation is introduced, including a double‐jointed camera at 12 o'clock position, monopolar scissors on the right 3 o'clock position, which is exchanged for a needle driver when suturing, and two extended‐range force bipolar forceps at the 6 o'clock and 9 o'clock positions while the ROSI is inserted directly through the SP access kit for the surgeon to perform autonomous suctioning, retraction and irrigation.

The EP space is further developed to expose the prostate. The puboprostatic ligaments, endopelvic fascia and the dorsal vascular complex (DVC) are kept intact to avoid damaging the continence mechanism. The superficial portion of the DVC is controlled with bipolar cautery. However, the preplacement of a DVC stitch, as described in Millin's technique of retropubic simple prostatectomy, is not required. The bladder neck is then incised erring more towards the prostate side anteriorly until the Foley catheter is identified. For a large median lobe, the incision is extended laterally to deliver the median lobe of the gland. The cobra‐down feature helps mimic the ‘30‐degree down’ view and better visualize the ureteral orifices, as well as the inferior extent of the median lobe.

The Foley catheter is then held on traction with the 9 o'clock instrument and an incision is then made on the posterior bladder neck. If a median lobe or intravesical protrusion is encountered or is anticipated preoperatively per cross‐sectional imaging, then this lobe may be either lifted directly with the 9 o'clock instrument, as the force bipolar forceps provide adequate grasp and upward traction using the *strong* feature or alternatively, the median lobe may be oversewn and elevated using a 2‐0 vicryl suture on a CT‐1 needle.

The mucosa is incised posteriorly at the distal third point in the case of a median lobe. The maintenance of a generous mucosal flap posteriorly allows an easier trigonization and performance of a vesicourethral anastomosis. Enucleation begins by incising the prostatic capsule and bluntly dissecting to the subcapsular adenomectomy plane. This plane is maintained with a combination of blunt and sharp dissection and using focal monopolar or bipolar cautery as needed to cauterize and control any vessels. Posterior dissection is completed at the level of the anatomical capsule followed by lateral, anterior and apical dissection. Given the limited retraction force of the SP robot and absence of a robotic tenaculum, dissection often entails toggling between posterior, lateral and anterior planes. The ‘strong’ feature of the force bipolar instrument may also be utilized to provide added retraction. Switching between the endoscope ‘Cobra’ to ‘Straightening’ feature may assist in posterior and lateral enucleation of the gland. Throughout the whole enucleation process, the prostatic capsule is maintained as the anatomical landmark. The anterior and apical dissection is performed last coming through the proximal most aspect of the anterior commissure where the convexities of the two lateral lobes merge in the midline, followed by a sharp urethral incision thus preserving an adequate safety margin from the sphincteric urethral complex and the anterior Retzius space structures including the DVC, puboprostatic ligament complex, endopelvic fascia and arcus tendineus.

The adenoma may be removed entirely en bloc especially in cases where the prostate volume is <200 cc and or in two to three pieces in case of a very large gland (>200 cc). Any small pieces of residual adenoma may be removed per the surgeon's discretion. Haemostasis is ensured with the use of bipolar cautery and use of 4‐0 vicryl or 3‐0 V‐Lok barbed suture to oversew any large venous channels or arterial bleeders. The specimen is placed in a retrieval bag. A mini‐lap sponge will be placed in the fossa during this step. Instruments are removed and the robot temporarily undocked, leaving wound retractor in place, and the specimen extracted. The access port kit is then reassembled, all instruments reinserted and the mini‐lap removed by the assistant. Similar to the TV technique, a 360° mucosal advancement flap is carried out in a running circumferential fashion using a 3‐0 V‐Lok suture on a V‐20 needle reapproximating the mucosa of the bladder neck to the remnant of the prostatic urethra. A circumferential anastomosis is preferred when technically feasible; however, in certain cases where the gap anteriorly is longer, the anterior prostatic capsule may be reapproximated to the anterior portion of the bladder neck using a barbed suture (not shown in video). CBI is not routinely required and patients may be discharged home the same day if discharge criteria, per surgeon preference are met.

## 4 RESULTS

A total of 35 consecutive patients successfully underwent SP EP RASP between July 2025 and April 2026. No operations required conversion to open or multi‐port approaches. Baseline patient demographics and clinical characteristics are summarized in Table 1. The mean patient age was 72.34 ± 6.73 years, with a mean BMI of 27.76 ± 5.07 kg/m^2^. The mean CCI was 3.06 ± 0.84, and the mean ASA score was 2.45 ± 0.56. All patients underwent surgery for BPH with LUTS refractory to medical management. Eleven patients (31.4%) had a history of at least one episode of acute urinary retention, and two patients (5.7%) had concomitant bladder stones. Preoperatively, 16 patients (45.7%) required an indwelling Foley catheter due to urinary retention, had elevated post‐void residual or a finding of bilateral hydronephrosis on imaging secondary to bladder outlet obstruction. Previous abdominal surgery was documented in 17 patients (48.5%), and five patients (14.2%) had a history of prior inguinal or pelvic surgery. Prior prostatic intervention was present in one patient (2.9%). The mean prostate volume on preoperative imaging was 122.42 ± 49.07 cc.

**TABLE 1:** Demographic and preoperative characteristics of the study population.

Variables	Single‐port extraperitoneal cohort (*n* = 35)
Age (year), mean (SD)	72.3 (6.7)
BMI (kg/m^2^), mean (SD)	27.8 (5.1)
CCI, mean (SD)	3.06 (0.84)
ASA physical status grading, mean (SD)	2.45 (0.56)
History of hypertension, *n* (%)	22 (62.9)
History of type 2 diabetes mellitus, *n* (%)	8 (22.9)
History of hyperlipidaemia, *n* (%)	23 (65.7)
History of prior inguinal/pelvic surgery, *n* (%)	5 (14.2)
History of prior abdominal surgery, *n* (%)	17 (48.5)
History of prior prostatic intervention, *n* (%)	1 (2.9)
History or presence of bladder stones, *n* (%)	2 (5.7)
History of urinary retention, *n* (%)	11 (31.4)
Preoperative Hgb, mean (SD)	13.67 (1.68)
Preoperative PSA (ng/mL), mean (SD)	7.21 (7.85)
Prostate volume (mL), mean (SD)	122.42 (49.1)
Preoperative biopsy done, *n* (%)	7 (20.0)
Malignancy on biopsy	1
Preoperative urinary retention requiring catheter, *n* (%)	16 (45.7)

Abbreviations: ASA, American Society of Anesthesiologists; BMI, body mass index; CCI, Charlson Comorbidity Index; Hgb, haemoglobin; IQR, interquartile range; PSA, prostate‐specific antigen.

### 4.1 Intraoperative outcomes

All 35 procedures were completed successfully using the SP EP approach without conversion to open or multi‐port techniques. Intraoperative, postoperative and histopathology outcomes are summarized in Table 2. The median operative time was 166 [156, 180] min, and the median console time was 132 [118, 148] min. Median EBL was 75 [50, 100] mL, with a mean haemoglobin change of −0.98 ± 0.64. No patients required intraoperative or perioperative blood transfusion (0%). A ‘plus one’ 5‐mm assistant port was utilized in 2 of 35 cases (5.7%). No air embolism events were observed. The median enucleation efficacy was 58.4% [50.0, 63.6] calculated by the ratio of the specimen weight to the estimated prostate volume on preoperative cross‐sectional imaging. Incidental malignancy was identified in seven patients (20%). En bloc enucleation was achieved in 31 of 35 cases (88.6%), while four cases (11.4%) required non‐en bloc (piecemeal) enucleation (see Table 3).

**TABLE 2:** Intraoperative, postoperative and histopathology outcomes associated with single‐port robot‐assisted simple prostatectomy (RASP) used in this study.

Variables	Single‐port extraperitoneal cohort (*n* = 35)
Intraoperative parameters	
Console time (min), median [IQR]	132 [118, 148]
Operative time (min), median [IQR]	166 [156, 180]
Estimated blood loss (mL), median [IQR]	75 [50, 100]
Conversion to multi‐port, *n* (%)	0 (0.0)
Drain placement, *n* (%)^a^	1 (2.9)
Transfusions given, *n* (%)	0 (0.0)
Haemoglobin change (g/dL), average (SD)	−0.98 (0.64)
Histopathology	
Specimen weight (g), median [IQR]	63.3 [46.0, 90.0]
Specimen weight/preoperative estimated weight, median% [IQR]	58.4 [50.0, 63.6]
Incidental malignancy finding, *n* (%)	7 (20)
Postoperative care	
Average length of stay (h), median [IQR]	7.5 [4.8, 24.0]
Same‐day discharge, *n* (%)	17 (49)
Discharged with narcotics, *n* (%)	1 (2.9)
Continuous bladder irrigation requirement, *n* (%)	0 (0.0)
Foley catheter duration (days), median [IQR]	8.5 [6, 10]
Postoperative outcomes	
30‐day postoperative complications, *n* (%)^b^	1 (2.9)
30‐day readmission, *n* (%)	1 (2.9)
Post‐void residual at follow‐up, average (SD)	24.03 (29.0)
Early continence at 4 weeks,^c^ *n*/*N* (%)	31/35 (88.6)
Continence at 3 months,^c^ *n*/*N* (%)	22/23 (95.7)

^a^ Drain placed due to prostatic abscess.

^b^ Epididymo‐orchitis requiring admission for IV antibiotics.

^c^ Continence defined as pad‐free status or use of a single pad for safety.

**TABLE 3:** Comparison of en bloc enucleation versus non‐en bloc enucleation for SP EP RASP.

Variables	Group	*n*	Mean (SD)	*p* value^a^
Preoperative estimated volume (cc)	Non‐en bloc enucleation	4	207.3 (29.3)	0.005
	En bloc enucleation	31	111.5 (39.5)	
Specimen weight (g)	Non‐en bloc enucleation	4	137.8 (44.7)	0.006
	En bloc enucleation	31	63.5 (30.8)	

^a^ Mann–Whitney *U* test used for comparison of each variable between groups; given *n* = 4 in the non‐en bloc group, this comparison is exploratory and should be interpreted with caution.

### 4.2 Postoperative outcomes

CBI was not required in any patients (0%). The median length of hospital stay was 7.5 [4.8, 24.0] h. SDD using all 35 patients as the denominator (raw rate) was achieved in 17 patients (49%), and the majority of patients were discharged within 24 h of surgery. Four patients required planned admission due to cardiac or anaesthesiology recommendations. Excluding these four planned admissions, the adjusted SDD rate was 54.8% (17/31). The remaining 14 patients requested to stay overnight in the hospital due to lack of postoperative support at home. A surgical drain was placed in one patient (2.9%) due to an intraoperative prostatic abscess encountered during the enucleation process. Only one patient (2.9%) required narcotic analgesia at discharge. The median Foley catheter duration was 8.5 days.

### 4.3 Complications

The overall 30‐day complication rate was 2.9% (1/35), consisting of a case of epididymo‐orchitis requiring hospital admission for intravenous antibiotics (Clavien–Dindo II). The 30‐day readmission rate was 2.9% (1/35). No major complications (Clavien–Dindo ≥III) were observed. No patients developed bladder neck contracture, urethral stricture or required reintervention during the follow‐up period.

### 4.4 Functional outcomes

Mean PVR at follow‐up was 24.03 ± 29.03 mL. Early continence, defined as pad‐free status or use of a single pad for safety, at 4 weeks was achieved in 88.6% patients (31/35), and continence at 3 months was achieved in 95.7% of patients with available follow‐up (22/23). De novo urinary incontinence occurred in four patients at 4 weeks, characterized as stress predominant in three and mixed in one, and was expectantly managed with pelvic floor physical therapy and anticholinergic medication. The three patients with stress‐predominant incontinence regained full continence by 3 months, while the patient with mixed incontinence regained full urinary control by the 6‐month visit.

### 4.5 Learning curve

**FIGURE 1:**
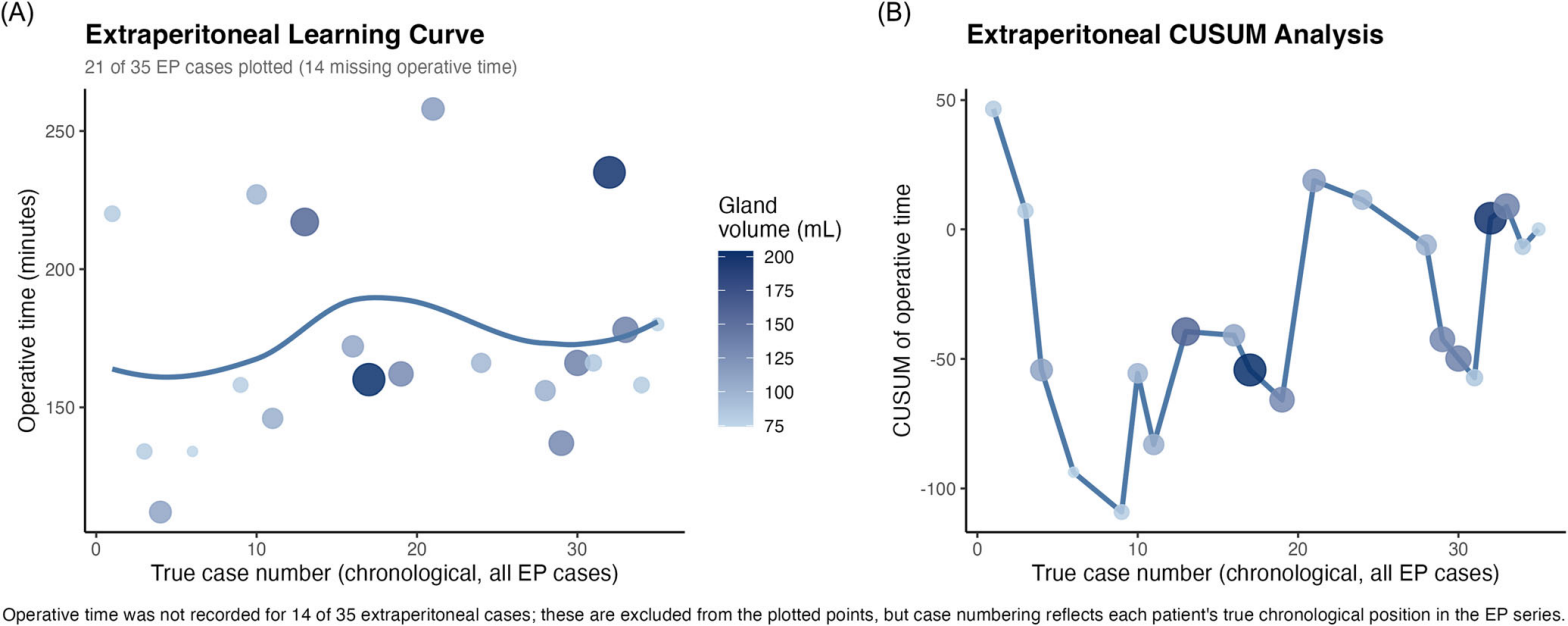
Learning curve (A) and cumulative sum (CUSUM) (B) analysis demonstrating operative time trends. Plotted sequentially for each case, ordered chronologically.

Operative time was not recorded for 14 of 35 EP cases; these were excluded from the learning curve and CUSUM analyses, and the remaining 21 cases are shown in Figure 1 at their true chronological position. CUSUM analysis demonstrated an early peak followed by fluctuation around the mean rather than a sustained plateau, and the LOESS curve similarly showed variability across the case range. Given the missing operative time data and overall small sample size, these findings are presented as exploratory.^5^

## 5 DISCUSSION

This series represents one of the first comprehensive reports of SP EP RASP as an alternative to the increasingly adopted TV approach. Our findings suggest that SP EP RASP is a safe and feasible technique for managing large volume BPH, with perioperative outcomes comparable to those reported for both TV SP and multi‐port approaches.^4^, ^6^, ^7^

The TV approach to SP RASP has gained significant traction since its description by Kaouk et al., with multiple series demonstrating excellent perioperative outcomes, high rates of SDD, minimal blood loss and low complication rates.^4^, ^6^, ^8^ The median operative time in our series was 166 min, which falls within the reported range of 107–180 min for TV series, acknowledging differences in surgeon experience and case complexity.^4^, ^6^, ^9^ Similarly, the median blood loss of 75 mL and median percentage of tissue enucleated of 58.4% in our cohort are comparable with the largest single‐institution case series described by Ramos et al., who reported a 100‐mL blood loss and median enucleation percentage of 60.7%.^4^ The adjusted rate of SDD in our cohort was 54.8% in comparison with the 78.6% rate of SDD reported by the Single‐Port Advanced Research Consortium.^3^ No air embolism events occurred in our cohort. This rare but potentially serious complication has been reported in TV approaches and is thought to be related to high pneumovesicum pressures in a valveless insufflation system. The EP approach inherently uses a larger working space obviating intravesical insufflation and thus, may avoid the mechanism implicated in air embolism formation, though this hypothesis needs to be further tested and validated.^4^

An extravesical approach to simple prostatectomy is not itself novel; Shahait et al. previously described a bladder neck sparing, extravesical RASP.^10^ Additionally, longstanding bladder outlet obstruction drives progressive bladder remodelling through cyclic stretch, elevated detrusor pressures and hypoxia.^11^ In a TV approach, the cystotomy, self‐retaining retractor and prolonged bladder‐wall traction may theoretically compound this baseline injury, a concern previously raised by the authors of the extravesical RASP technique.^10^ By approaching the adenoma extravesically, the EP technique avoids a cystotomy and reduces detrusor traction and manipulation though this rationale is mechanistic and was not tested in the present series.

From a technical standpoint, the EP technique facilitated a high rate of en bloc enucleation (88.6%), with only four cases requiring piecemeal resection. These four cases had a mean specimen weight of 137.8 g and a mean preoperative prostate volume of 207.25 cc consistent with a higher likelihood of piecemeal enucleation in very large glands; however, given the small subgroup, this association is descriptive and exploratory. The larger EP working space may facilitate en bloc enucleation once the learning curve is overcome. The operating surgeon found the wider panoramic exposure particularly helpful during anterior and apical dissection, in preserving key anterior Retzius space structures such as the sphincteric urethral complex and in reducing unintended venous bleeding secondary to improper surgical plane selection.^12^ The da Vinci SP extended‐range force bipolar instruments offer a better grip and stronger retraction than conventional standard range instruments but require greater working space for optimal retraction. These extended‐range instruments in the conventional TV approach may be somewhat limited by the smaller working space within the bladder, whereas the EP approach may optimize their use, especially for retraction during enucleation.

Our outcomes are descriptively similar to those reported for multi‐port RASP series. A previously described comparison of the SP TV RASP and multi‐port RASP by Abou Zeinab et al. showed significantly lower blood loss (100 vs. 150 mL; *p* < 0.001) and shorter median LOS (17.7 vs. 33 h, *p* < 0.001) in the SP Cohort, with CBI used less frequently in SP RASP than in multi‐port RASP (17.3% vs. 64.4%, *p* < 0.001).^3^ In line with these results, our cohort demonstrated an EBL of 75 mL, no CBI use, a median LOS of 7.5 h and an overall adjusted SDD rate of 54.8% comparable with multi‐port RASP series with similar reported mean blood loss and catheter duration.^3^, ^7^ Relative to endoscopic enucleation, contemporary propensity‐matched comparisons of RASP and en bloc holmium laser enucleation of the prostate (HoLEP) have reported broadly similar overall success, with HoLEP favouring shorter operative and catheterization times and RASP favouring shorter hospital stay and lower rates of early SUI.^13^, ^14^ The regionalized nature of the EP approach avoids the peritoneal cavity altogether, which may theoretically decrease the risk of bowel‐related complications such as ileus and may ultimately translate into shorter LOS, decreased postoperative pain and potentially faster convalescence.^7^, ^12^, ^15^

The overall 30‐day complication rate was 2.9%, with no major (Clavien–Dindo ≥III) complications; the sole event was epididymo‐orchitis requiring intravenous antibiotics, which resolved fully. Avoidance of the peritoneal cavity may broaden applicability to patients with prior major abdominal surgery, and the bladder neck‐to‐urethra reconstruction may obviate CBI and may mitigate irritative voiding symptoms. No bladder neck contractures or urethral stenosis occurred, though follow‐up is short‐term. Our reported median Foley catheter duration of 8.5 days is notably longer than that reported in contemporary SP TV series and was driven by postoperative provider scheduling availability rather than patient‐specific clinical factors.

The rate of incidental prostate cancer in our cohort was 20%, occurring in patients who would not have qualified for PSA screening given advanced age, as well as in patients with prior negative MRI, normal PSA values or negative prior biopsies. While this is higher than the <3% reported in large contemporary administrative datasets of mixed BPH surgical procedures, and the ~1% to 2% reported for RASP within those datasets, such datasets are dominated by transurethral resection, which samples only a portion of the gland.^16^ Reported rates of incidental prostate cancer following HoLEP—a procedure analogous to ours in the volume of tissue removed—range widely from approximately 5.6% to 23.3%.^17^, ^18^ Notably, a large contemporary HoLEP series of 913 heavily prescreened patients reported a 20% incidence of incidental prostate cancer, with affected patients similarly having had negative preoperative MRI or prior negative biopsy in a substantial proportion of cases.^17^ Our finding is therefore consistent with the upper range of contemporary enucleation series rather than anomalous and likely reflects both the completeness of adenoma removal afforded by the EP approach and an older patient population, several of whom were beyond the age at which PSA screening is routinely recommended. Patients with incidental malignancy have either elected active surveillance or watchful waiting after shared decision‐making and are being monitored with PSA surveillance.

Functional outcomes in this series were favourable, with a mean postoperative PVR of 24.03 mL. All patients in our cohort with preoperative urinary retention requiring an indwelling Foley passed a successful trial of void. Among evaluable patients with available follow‐up, continence was achieved in 31/35 patients (88.6%) at 4 weeks and 22/23 (95.7%) patients at 3 months. The lower denominator at 3 months reflects shorter follow‐up for patients treated later in the study period rather than clinical attrition. De novo stress incontinence occurred in four patients at the 4‐week follow‐up and was managed expectantly with pelvic floor physical therapy or anticholinergic medical therapy; of these, three regained full urinary control by 3 months. These outcomes fall within the range reported in published TV series, recognizing differences in study design, surgeon experience and follow‐up.^9^, ^19^ Continence preservation likely reflects precise apical and anterior dissection and performance of the commissurotomy at the proximal convexity where the two lateral lobes merge anteriorly, maintaining a safe distance from the sphincteric urethral complex.^12^ These data are reassuring but should be interpreted as preliminary, and longer follow‐up with standardized patient‐reported urinary outcomes remains necessary. To date, no patient has required a salvage continence procedure such as a midurethral sling or artificial urinary sphincter.

The EP approach offers several practical advantages. It provides a familiar workspace for surgeons experienced with retropubic or extraperitoneal pelvic surgery, potentially facilitating adoption.^12^, ^20^ It is well suited for patients with prior abdominal or pelvic surgery; in our cohort, all cases were completed without conversion despite 48.5% of patients having prior abdominal surgery. No patient required CBI, and only 2.9% (1/35) were discharged with narcotic analgesia. Finally, the supine position without Trendelenburg may benefit medically complex or obese patients by minimizing cardiopulmonary strain.^12^

Our series included patients with large estimated prostate volumes (mean 122.42 cc, SD 49.1 cc). The learning curve analysis characterized the surgeon's initial independent experience. CUSUM analysis identified an early peak followed by a quick drop and fluctuation around the mean rather than a sustained plateau, and the LOESS curve similarly showed variability across the range, though the surgeon had performed over 100 prior SP urologic procedures. Consistent anatomic landmarks and avoidance of intravesical dissection may facilitate skill acquisition, particularly for surgeons experienced with extraperitoneal and retropubic techniques.^12^, ^20^ Residual variability in operative time likely reflects case‐mix factors including gland size, body habitus and prior abdominal surgery.

This study has several limitations. It represents a single‐surgeon, single‐institution experience, which may limit generalizability. The sample size of 35 patients is relatively small, and follow‐up duration is limited, precluding assessment of long‐term durability and late complications. Interpretation of enucleation efficacy is limited by heterogeneity in prostate volume measurements captured by different imaging modalities and outside radiologists' interpretation; imaging modality was not standardized. Although early continence outcomes were encouraging, these findings should be interpreted cautiously given the relatively short duration of follow‐up and incomplete availability of long‐term functional assessment; 3‐month continence data were available for 23/35 patients at the time of submission. Postoperative maximum flow rate (*Q*_max_) and IPSS were not part of our standard postoperative follow‐up protocol and are not reported; PVR is routinely collected and is reported above. Standardized postoperative patient‐reported outcome measures including IPSS, quality‐of‐life scores, irritative voiding symptoms, ejaculatory function and SHIM scores were not systematically collected in this early experience and represent important limitations of the present study.

## 6 CONCLUSION

SP EP RASP is a technically feasible approach that preserves the benefits of regionalized surgery while affording a larger working space facilitating anterior and apical prostate adenomectomy, with favourable early perioperative outcomes in this initial series. The described technique demonstrates clinically insignificant blood loss, decreased morbidity in patients with hostile abdomen or cardiopulmonary compromise, satisfactory rates of SDD, elimination of CBI and demonstrates early return of continence. Future prospective studies with standardized patient‐reported outcomes, comparative analyses to endoscopic and SP TV techniques, and longer term follow‐up are needed to better define the role of this technique for the surgical management of BPH.

## AUTHOR CONTRIBUTIONS

Sij Hemal conceived and developed the surgical technique, performed the surgical procedures, curated and interpreted the clinical data and drafted the manuscript. Dr. Hemal also edited the surgical video presented here and provided the script of the narration. Samantha H. Rosen, Simon Kim, Jacob S. Hershenhouse and Joon Yau Leong contributed to data collection, statistical evaluation and assisted in critical revision of the manuscript for important intellectual content, reviewed and approved the final version and agreed to be accountable for the work. Samantha H. Rosen also contributed to the narration of the technique drafted by Dr. Hemal and also in the submission process. Michael Eppler and Patrick Ford contributed to the revision of the manuscript.

## DISCLOSURE

AI‐assisted writing tools were used solely for language refinement, to improve grammar, sentence clarity and editorial flow. All data collection, analysis, clinical interpretation and intellectual contributions to the manuscript are entirely the work of the research team.

## CONFLICT OF INTEREST STATEMENT

No conflicts exist.

## Supporting information


**Data S1.** Supporting Information.

**Movie S1.** Supporting Information.

**Video S1.** Supporting Information.

## Data Availability

The data that support the findings of this study are available on request from the corresponding author. The data are not publicly available due to privacy or ethical restrictions.
