# Supplementary material for: Single‐port extraperitoneal robot‐assisted simple prostatectomy for benign prostatic hyperplasia: Surgical technique and initial clinical experience
Source: BJUI Compass. 2026 Aug 24;7(8):e70273. doi: 10.1002/bco2.70273 (PMC13504154; doi:10.1002/bco2.70273)
Supplement: Supplementary file 1 [file BCO2-7-e70273-s001.docx]

Before beginning the video:

Introductory Slide:

00:00: After obtaining extraperitoneal access the steps of which are described the robotic view is shown. The prostate has already been defatted and the space of Retzius dissected further to identify the pubic bone and further create the extraperitoneal space. The puboprostatic ligaments, dorsal venous complex and the endopelvic fascias are left untouched.

With the camera in the “Cobra Down” position, the bladder neck will then be incised and dissection will be carried down to the foley catheter. In cases where a median lobe or an intravesicular protrusion is expected based on preoperative imaging, the bladder neck incision may be extended laterally to adequately deliver the median lobe. In this particular case, the patient did not have a median lobe.

00:20: The foley catheter is then lifted on traction and the posterior bladder neck incision is carried out following which the posterior enucleation will be performed after identifying the subcapsular adenomectomy plane. Using blunt and sharp dissection we carry out this plane posteriorly first.

00:56: the endoscope may be straightened in order to gain better visualization of the posterior enucleation plane and furthermore optimize upward traction with the 9’o clock instrument. Enucleation is carried out posteriorly as distally as possible using blunt dissection as well as cauterizing any small capsular vessels with monopolar or bipolar cautery.

1:17: now we begin the lateral enucleation plane with care taken to stay in the shiny white adenoma plane.

2:10: At this point we switch to the anterior/anterolateral dissection. This is the part where our technique is very beneficial in visualizing the anterior/apical portion of the adenoma and carefully developing this plane under good visualization. Toggling between the endoscope straighten/cobra down position may be especially advantageous in visualizing the apical extent of the adenoma and avoid any perforation of the anterior capsule and the dorsal vascular complex.

3:04: We then come through the anterior commissure at the level where the two lateral lobes merge in the midline. It is important to make the incision at this proximal convexity in order to preserve the length of the prostatic urethra for performing the reconstruction and to avoid any inadvertent injury to the external urinary sphincter.

3:36: The prostate may be rotated after the commissurotomy in order to better recognize the interface between the prostatic capsule and the adenoma. It is extremely important to have this plane pre-developed in order to avoid an inadvertent capsulotomy while removing the adenoma.

3:50: The en bloc specimen is bagged and removed by the assistant. A sponge may be placed in the pelvic cavity to resorb any blood products or urine.

4:00 Here we will demonstrate the steps to perform the procedure in the case of a median lobe. In this first example the median lobe is oversewn with a 2-0 vicryl suture on a CT-1 needle and retracted upwards. A mucosal incision is then made on the distal part of the median lobe and the enucleation plane is developed beginning at the median lobe and carrying this plane posteriorly to perform the posterior enucleation step.

5:01: In this example a larger median lobe can be seen that can also be lifted with the 9’o clock fenestrated bipolar instrument using the strong feature. Similar, to our index procedure the enucleation steps can be carried out. In this example the lateral enucleation was performed first in order to obtain more leverage posteriorly in order to obtain adequate upward traction on the bulky posterior lobe. The larger glands can also be enucleated as a whole or in a piecemeal fashion per surgeon preference.

6:05: Now we return to our index case. A complete vesicourethral anastomosis will now be carried out using a 3-0 V-Lok suture on a V-20 needle. The second suture used will be a 3-0 V-Lok suture on a CV-23 needle. The preservation of the muscosa on the median lobe helps to perform this anastomosis.

7:00 The redundant anterior bladder neck may be closed in an anterior tennis racket fashion. The anastomosis will then be checked with 120 cc of saline to ensure water tightness.

7:11: After checking the anastomotic integrity the anterior prostatic capsule can be reapproximated to the anterior perivesical tissues in a running fashion using a 3-0 V-Lok suture to help complete a total anatomic reconstruction.

7:30 Conclusion slide
